# Long-term outcome of acromioclavicular joint injuries in an urban population

**DOI:** 10.1136/bmjsem-2025-003183

**Published:** 2026-11-10

**Authors:** Stein Arve Skjaker, Martine Enger, Andreas Torgalsen, Are Hugo Pripp, Lars Nordsletten, Berte Bøe

**Affiliations:** 1 Division of Orthopaedic Surgery, Oslo University Hospital, Oslo, Oslo, Norway; 2 Institute of Clinical Medicine, University of Oslo, Oslo, Oslo, Norway; 3 Department of Orthopaedic Surgery, Akershus University Hospital, Lørenskog, Akershus, Norway; 4 Oslo Centre of Biostatistics and Epidemiology, Research Support Services, Oslo University Hospital, Oslo, Oslo, Norway

**Keywords:** Arthroscopy, Shoulder, Sports medicine, Collar bone

## Abstract

**Objective:**

To assess long-term outcomes following acromioclavicular (AC) joint injuries and associations between injury severity and patient-reported shoulder function.

**Methods:**

This prospectively registered cohort included all patients diagnosed with an AC joint injury at Oslo University Hospital in 2013–2014. Participants were invited to follow-up in 2021. Patient-reported outcome measures (PROMs) included the Oxford Shoulder Score (OSS), Quick Disabilities of Arm, Shoulder and Hand, and Numeric Rating Scale for shoulder pain. A subset underwent clinical examination using the Constant-Murley Score, shoulder tests and anteroposterior radiographs. Radiographs were evaluated for Rockwood classification and degenerative changes.

**Results:**

A total of 240/287 (84%) participants responded at a mean follow-up of 7.6 years (SD 0.3). Among respondents, 72% had Rockwood type I or II injuries, 24% had type III injuries and 4% had type V injuries. The mean OSS was 41.8 (SD 8.6) of 48, without significant differences comparing Rockwood type I with types II, III or V injuries (p>0.05). Younger females aged 20–30 and males aged 20–40 recorded lower OSS scores than normative data (p<0.05). 18 participants were classified as OSS outliers, mainly Rockwood types I and III. Six respondents (3%) had undergone AC surgery. Radiographic degenerative changes were prevalent (96 out of 150, 64%) but did not correlate with functional outcomes.

**Conclusion:**

In this unselected urban cohort, most respondents reported favourable long-term shoulder function, although some experienced ongoing symptoms. OSS did not differ significantly between Rockwood categories, although comparisons involving Rockwood type V were limited by small sample size. Radiographic degeneration was common but unrelated to PROMs.

**WHAT IS ALREADY KNOWN ON THIS TOPIC**

- Non-operative treatment is the preferred initial approach for Rockwood type I–II injuries and is commonly recommended for type III injuries. Increasing evidence also supports initial non-operative management for selected high-grade injuries, although uncertainty remains.

**WHAT THIS STUDY ADDS**

- In this unselected urban cohort, most respondents reported favourable long-term shoulder function. Oxford Shoulder Score (OSS) did not differ significantly between Rockwood types I, II, III and V, although comparisons involving Rockwood type V injuries were limited by small sample sizes.

**HOW THIS STUDY MIGHT AFFECT RESEARCH, PRACTICE OR POLICY**

- Patients with low-grade acromioclavicular (AC) joint injuries can generally be reassured regarding long-term function after initial non-operative management. Persistent pain or stalled recovery should prompt assessment for associated soft-tissue pathology rather than attribution of symptoms solely to the AC joint or immediate consideration of surgery.

## Introduction

 There are no evidence-based guidelines for the diagnosis and treatment of acromioclavicular joint (AC) joint injuries.^1–7^ The most widely used radiographic classification for AC joint injuries is Rockwood’s (table 1).^1 8^ However, long-term outcome data on non-operatively treated AC joint injuries are rare, aside from studies focusing on specific subgroups.^9 10^ Current consensus recommendations suggest a non-operative approach for Rockwood type I–III injuries.^1 3 11^ Follow-up of operative versus non-operative treatment does not show superior long-term outcomes with surgery for AC joint injuries, even for type V injuries.^12–17^ Additionally, operative treatment carries potential surgical complications and a prolonged recovery period.^3^

**Table 1:** Rockwood classification of acromioclavicular joint injuries on radiographs*

Rockwood type	CC distance	AC joint
I	Normal	Normal
II	0–25%	Normal or slightly elevated, and may be normal or widened AC joint
III	>25–100%	Lateral clavicle elevated above the superior border of the acromion
IV	Increased or normal CCD	Posterocranial displacement of the lateral clavicle
V	>100–300%	Lateral clavicle is grossly elevated above the acromion
VI	Reversed CC distance	Inferior AC dislocation

* Rockwood CA. Subluxations and dislocations about the shoulder: Injuries to the acromioclavicular joint. In: Rockwood CA, Green DP, eds. *Fractures and dislocations*. J.B. Lippincott; 1984:860–910.

AC, acromioclavicular; CC, coracoclavicular; CCD, coracoclavicular distance.

There is a lack of evidence-based knowledge regarding the long-term significance of AC joint injuries on chronic shoulder pain and function. The natural history of these injuries in the general population, in terms of patient symptoms and functional outcomes, has not been previously published to our knowledge. An epidemiological overview of shoulder injuries during 1 year in 2013–2014 noted that patients with AC joint injuries were typically young and active individuals (often injured during sports).^18 19^ Such injuries can therefore have a considerable impact in terms of sick leave, shoulder pain, functional impairment and quality of life for affected patients. Indeed, restricted shoulder movement and pain are major causes of morbidity and work absence.^20^

The sports medicine literature has highlighted the need for robust studies evaluating the natural history and long-term outcomes of non-operatively managed AC joint injuries.^21^

The present study aims to address this gap by providing long-term outcome data for a 1-year cohort of AC joint injuries in a general population, including the patients’ symptoms and objectives. We hypothesised that there was a significant difference in Oxford Shoulder Score (OSS) between injured patients and normative data at follow-up, as well as between Rockwood type I and types II, III and V AC joint injuries.

## Materials and methods

### Study design and population

A prospectively collected cohort of patients from the Department of Orthopaedic Emergency at Oslo University Hospital (OUH) was used for a follow-up study. The Department of Orthopaedic Emergency is the only public emergency department in Oslo dedicated to treating acute injuries, serving a population of approximately 630 000 as of 2013. An estimated 86–90% of the population would attend the facility in the event of an acute injury to the upper extremity.^22 23^ Among 2650 Oslo residents with a shoulder injury registered between May 2013 and April 2014, 287 patients had an AC joint injury (online supplemental figure 1).^18 19^ Patients who presented within 3 months of an acute injury and reported anterosuperior shoulder pain, along with a maximum point tenderness at the AC joint, were included. They were evaluated using non-weight-bearing anteroposterior (AP) radiographs, as per the OUH internal protocol. Their injuries were categorised following the Rockwood classification system (types I–VI).^8 24 25^

Follow-up assessments were conducted approximately 7–8 years post-injury in May and June 2021. Patients received an invitation letter 1 month prior to the follow-up appointment at the Department of Orthopaedic Emergency at OUH. They were asked to complete electronic questionnaires regarding their current shoulder status. A couple of days before the follow-up, they were reminded of their appointment and the electronic questionnaires. If they did not respond or attend, they received a Short Message Service with a new link to the electronic questionnaires.

### Person-reported outcome measures and objectives

At follow-up, patients were asked to fill in the primary outcome measure OSS, which ranges from 0 to 48 (higher scores indicate better function). Secondary, patient-reported outcome measures (PROMs) included the Quick Disabilities of Arm, Shoulder and Hand (QuickDASH, which ranges from 0 (best) to 100 (worst)), and Numeric Rating Scale (NRS) pain score (which ranges from 0 (best) to 10 (worst)) for shoulder pain over the prior 2 weeks.^26–35^

All patients attending the outpatient clinic underwent a clinical shoulder examination performed by an orthopaedic surgeon (AT or SAS). This examination included the Constant-Murley Score (CMS) for both the injured and non-injured sides (either physio IB or doctor SAS), as well as specific shoulder tests (either doctors AT or SAS) such as the following: AC joint pain palpation, asymmetric AC joints, tangent sign, Paxinos sign, O’Brien’s test, cross-body adduction test, Hawkins impingement test and AP radiographs.^36–38^ The CMS is a 0–100-point scale composed of several individual parameters, including pain, activity of daily living, mobility and strength, where a total score of 100 is the best possible. We also included an anamnestic questionnaire (online supplemental appendix A).

### Radiographic measurements and classification

The Rockwood classification at the time of injury was established first after a majority agreement (≥4/6) in the first round of the reliability study on the same cohort (n=219).^25 39^ Second, it was established by majority opinion in the first round (n=27). Third, there was total majority agreement (≥7/12) in both rounds from the reliability study (n=14). Fourth, it was established by total majority opinion in both rounds (n=10). Two radiographs remained difficult to categorise, and five were missing.

At follow-up, the patients (n=150) underwent standardised non-weight-bearing bilateral and unilateral Zanca radiographs, as per the OUH standard protocol.^25^ The unilateral radiographs of the injured shoulder were performed by tilting the X-ray beam 15° in the cephalic and caudal direction in a standing position. The radiographic evaluation involved measuring the shortest coracoclavicular (CC) distance and the AC height distance (D), on both the injured and uninjured sides (online supplemental figure 2). The AC width was measured from the mid-acromion to the mid-clavicular articular surface. All measurements were in millimetres (mm). Additionally, we gauged the height of the acromion (A) and calculated the D/A ratio as described by Gastaud *et al*.^40^ We evaluated the AC joint for post-traumatic changes, including osteophytes, distal clavicle osteolysis, AC joint arthrosis, ligament calcifications and other degenerative signs.^9 15 41–46^ The first author (SAS) reviewed all the follow-up radiographs.

### Statistical analysis

Descriptive statistics are presented as means with SD or as medians with IQR for continuous variables and as counts (%) for categorical variables. Continuous variables were compared using Student’s t-test or the Mann-Whitney U test, as appropriate, and categorical variables using the χ^2^ test or Fisher’s exact test when expected cell counts were small. In response to the distribution of injury severity, we also conducted a post hoc comparison between low-grade injuries (Rockwood types I and II) and high-grade injuries (Rockwood types III and V). To assess potential non-response bias, baseline characteristics were compared between responders and non-responders. The few operated patients (n=6) were analysed within the same group as the others. We conducted univariable and multivariable linear regression analyses, with OSS as the dependent variable and selected independent variables. For the Rockwood classification, we used type 1 as the largest group for reference. Data analyses were performed using Stata/SE (V.18.0, StataCorp, College Station, Texas, USA).

### Equity, diversity and inclusion statement

The author group is gender-balanced and includes junior, mid-career and senior researchers from various disciplines; however, all members are from the same country. Our study population consisted of individuals of all ages, including males and females, from diverse socioeconomic backgrounds in the city of Oslo. We believe our data from this large urban, long-term follow-up study can be generalised to most settings.

## Results

Of the 287 patients in the initial cohort, 240 (84%) responded to the follow-up questionnaire for the OSS, resulting in a 16% loss to follow-up (n=47) (table 2).

**Table 2:** Follow-up characteristics of patients with acromioclavicular joint injury, number (%) unless otherwise stated

	Total	Types I–III	Type V	P value
N	240	230	10	
Age at follow-up, years, median*	39.6 (19.7)	39.6 (19.6)	40.3 (16.0)	0.71
Male sex	198 (83)	189 (82)	9 (90)	0.52
NRS at injury† mean±SD	5.7±1.9	5.8±1.9	3.8±2.3	0.02
Sport injury	134 (56)	130 (57)	4 (40)	0.3
Right-sided AC joint injury	130 (54)	125 (54)	5 (50)	0.79
Electronic questionnaires				
Oxford Shoulder Score‡, mean±SD	41.8±8.6	41.8±8.5	41.4±10.3	0.89
QuickDASH§, mean±SD	8.2±12.6	8.3±12.8	5.9±7.1	0.56
NRS† at follow-up, mean±SD	1.2±1.8	1.2±1.8	0.9±0.9	0.64
Satisfaction with shoulder¶, mean±SD	7.4±2.8	7.4±2.8	6.6±3.2	0.36
Operated AC joint	6 (3)	5 (2)	1 (10)	0.14
Still use painkiller for AC joint	7 (3)	7 (3)	0 (0)	0.58
Smoker (since injury)	72 (30)	68 (30)	4 (40)	0.5
Clinical examination, n	161	157	4	
CMS (injured side)**, mean±SD	87.1±11.5	87.0±11.6	90.5±6.4	0.55
CMS (non-injured side)**, mean±SD	89.0±11.8	88.9±11.9	93.8±1.9	0.42
Positive shoulder tests:				
Pain AC joint palpation	21 (13)	21 (13)	0 (0)	0.43
Asymmetric AC joints	62 (39)	59 (38)	3 (75)	0.13
Tangent sign	34 (21)	31 (20)	3 (75)	0.01
Paxino’s sign	10 (6)	10 (6)	0 (0)	0.6
O’Brien’s test	36 (23)	36 (23)	0 (0)	0.28
Cross-body adduction test	12 (7)	12 (8)	0 (0)	0.57
Hawkin’s impingement test	33 (21)	31 (20)	2 (50)	0.14

* Data presented as median (IQR).

† NRS, which ranges from 0 (best) to 10 (worst). At injury as an indication for the intensity of pain, and at follow-up the pain in the shoulder for the past 2 weeks.

‡ Oxford Shoulder Score, which ranges from 0 (worst) to 48 (best).

§ QuickDASH, which ranges from 0 (best) to 100 (worst).

¶ Score, which ranges from 0 (worst) to 10 (best).

** CMS (0–100), which ranges from 0 (worst) to 100 (best).

AC, acromioclavicular; CMS, Constant-Murley Score; NRS, Numeric Rating Scale; QuickDASH, Quick Disability of Arm, Shoulder, and Hand.

The respondents had a median age of 39.6 years (IQR, 19.7 years), and the mean time to follow-up was 7.6 years (SD 0.3). They were predominantly male (83%). Among the 235 respondents with an available baseline Rockwood classification, 146 (62 %) had type I, 23 (10 %) type II, 56 (24 %) type III and 10 (4 %) type V injuries. The mean OSS was 41.8 (SD 8.6), with no significant differences (p=0.89) between type I–III and type V (mean±SD, 41.8±8.5 vs. 41.4±10.3, respectively). Regarding OSS, when compared with Rockwood type I (mean OSS 41.2±SD 9.4), no significant differences were found for type II (44.5±SD 4.0; p=0.10), type III (42.1±SD 7.5; p=0.55) or type V (41.4±SD 10.3; p=0.95). In an additional analysis, patients with Rockwood type I–II injuries were compared with those with type III–V injuries. The mean OSS was 41.7 (SD 8.9) in the type I and II group and 42.0 (SD 7.9) in the type III and V group, with a mean difference of 0.3 points (95% CI −2.2 to 2.8; p=0.81).

Baseline characteristics were generally similar between responders and non-responders (online supplemental table 1). There were no statistically significant differences in age, sex, pain intensity at injury, sport-related injury, side of injury or Rockwood classification. Sport-related injuries were less frequent among non-responders than responders (40% vs 56%), although the difference did not reach statistical significance (p=0.053). Rockwood classification was available for 45 of the 47 non-responders. Of these, 27 (60%) had type I injuries, 6 (13%) type II, 9 (20%) type III and 3 (7%) type V. This distribution did not differ significantly from that of responders (p=0.66).

A total of 18 outliers were noted; 13 in Rockwood type I, 4 in type III and 1 in type V (figure 1). The outliers did not differ significantly from the others in terms of age (p=0.92) and sex (p=0.55), but they experienced significantly more pain over the past 2 weeks on the NRS (mean 3.1±SD 3.3 vs 1.0±SD 1.5, respectively; p<0.01).

**Figure 1:**
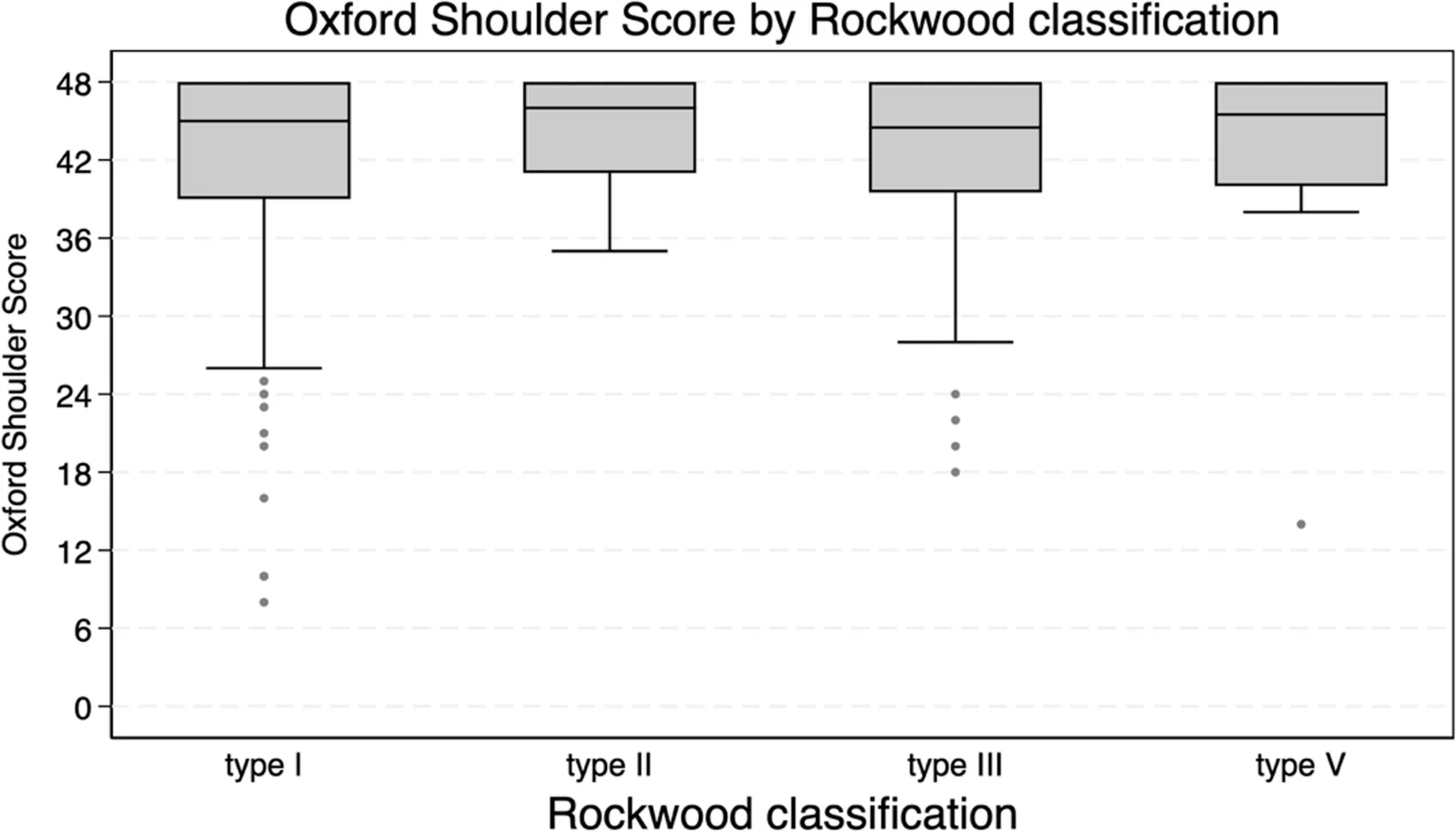
Box plot of Oxford Shoulder Score (OSS) by type of Rockwood classification. OSS, which ranges from 0 (worst) to 48 (best). The centre line of OSS in each box indicates the median (50th percentile) value for each Rockwood classification. The box represents the IQR from the 25th to the 75th percentile of OSS. The lower whisker extends to the 25th percentile minus 1.5 times the IQR. The upper whisker reaches up to the 75th percentile plus 1.5 times the IQR, but is not visible on our graph because the OSS score matches the upper 3rd quartile (75th percentile). Outliers are individual data points that lie far outside the main distribution and are represented by dots extending beyond the whiskers.

Of the six operated AC joints, two were type I, one was type II, two were type III and one was type V. None of these injuries were operated on at the Department of Orthopaedic Surgery, OUH. The type V and 1 of the type II were operated on in a chronic phase with biological reinforcement, while one type III was operated on early with an AC DogBone, which was later removed, all at private clinics. Both types I were operated on with capsule repair. A total of six (3%) were also operated on for other shoulder conditions during the same period. For instance, one patient with cuff repair of the supraspinatus and subscapularis underwent surgery at OUH in 2014, and two patients with superior labrum anterior to posterior injuries were operated on at a private clinic between 2016 and 2017.

Of those who answered the electronic questionnaires, 161 underwent a clinical examination with CMS (table 2). Regarding CMS on injured shoulder, when compared with Rockwood type I (mean CMS 86.7±SD 11.8), no significant differences were found for type II (85.5±SD 9.8; p=0.71), type III (88.5±SD 11.4; p=0.41) or type V (90.5±SD 6.4; p=0.53). Younger females (ages 20–30) and males (ages 20–40) had significantly lower OSS scores compared with the normative population (p<0.05) (tables 3 and 4).

**Table 3:** The mean Oxford Shoulder Score (OSS)* and SD for each age group in females, compared with a normal population†

	Clement *et al*†		Current study			
Age group (y)	N	OSS (SD)	N	OSS (SD)	Diff (95% CI)	P value
10–19			6	44.5 (3.1)		
20–30	11	46.9 (1.4)	7	41.9 (6.8)	−5.0 (−9.4 to −0.6)	0.03
31–40	17	44.3 (2.5)	14	41.1 (11.3)	−3.2 (−9.0 to 2.6)	0.26
41–50	8	45.4 (2.9)	9	41.1 (8.2)	−4.3 (−10.8 to 2.2)	0.18
51–60	12	41.7 (3.7)	5	35.4 (8.9)	−6.3 (−12.6 to 0.0)	0.05
61–70	15	39.7 (3.8)	1	48 (0)	8.3	

* Dawson J, Rogers K, Fitzpatrick R, Carr A. The Oxford shoulder score revisited. *Arch Orthop Trauma Surg*. Jan 2009;129(1):119–123. doi:10.1007 /s00402-007-0549-7.

† Clement ND, Court-Brown CM. Oxford shoulder score in a normal population. *Int J Shoulder Surg*. Jan 2014;8 (1):10–14 (adjusted to an OSS 0–48 scale, 48 best score).

**Table 4:** The mean Oxford Shoulder Score (OSS)* and SD for each age group in males, compared with a normal population†

	Clement *et al*†			Current study		
Age group	N	OSS (SD)	N	OSS (SD)	Diff (95% CI)	P value
10–19			19	40.1 (11.3)		
20–30	15	47.1 (1.1)	81	42.9 (7.8)	−4.2 (−8.2 to −0.2)	0.04
31–40	11	46.4 (2.0)	44	40.0 (9.4)	−6.4 (−12.1 to −0.6)	0.03
41–50	14	46.4 (2.2)	26	44.4 (4.9)	−2.0 (−4.8 to 0.8)	0.16
51–60	12	43.1 (4.1)	19	41.7 (8.5)	−1.4 (−6.8 to 4.0)	0.6
61–70	13	42.2 (4.1)	6	38.3 (14.4)	−3.9 (−12.8 to 5.0)	0.37
71–80	15	42.5 (5.0)	2	37.0 (2.8)	−5.5 (−13.3 to 2.3)	0.16

* Dawson J, Rogers K, Fitzpatrick R, Carr A. The Oxford shoulder score revisited. *Arch Orthop Trauma Surg*. Jan 2009;129(1):119–123.

† Clement ND, Court-Brown CM. Oxford shoulder score in a normal population. *Int J Shoulder Surg*. Jan 2014;8 (1):10–14 (adjusted to an OSS 0–48 scale, 48 best score).

17 individuals with type I, 1 individual with type II, 4 individuals with type III and no individuals with type V had an NRS score of 4 or higher (online supplemental table 2).

In univariate and multivariate analyses (R^2^=0.02), we did not identify any determinants associated with the OSS score (online supplemental table 3).

Among all individuals with a radiograph at follow-up, 64% (n=96) had a degenerative finding (table 5). None of the degenerative findings, such as joint osteophytes, arthrosis, osteolysis, calcifications of the coracoclavicular ligament, or joint deformity, or the absence of these, was associated with a change in OSS in a regression model. The mean CC distance on the injured versus uninjured side was 8.0 mm versus 7.9 mm for type I, 8.9 mm versus 8.5 mm for type II, 11.3 mm versus 7.1 mm for type III and 15.3 mm versus 6.3 mm for type V.

**Table 5:** Findings in (n=150) individuals’ radiographs at 7.6-year follow-up of AC joint injury, number (%) unless otherwise stated

Rockwood	
Type I	109 (73)
Type II	19 (13)
Type III	16 (11)
Type V	6 (4)
AC joint osteophytes	80 (53)
AC joint arthrosis	71 (47)
AC joint osteolysis	12 (8)
AC joint deformity	30 (20)
CC ligament calcification	19 (13)
No degenerative AC joint	54 (36)
Change in Rockwood type from injury classification to follow-up:	
Reduced by 2	5(3)
Reduced by 1	8(5)
Same as first time	102 (69)
Increased with 1	19 (13)
Increased with 2	14 (9)

AC, acromioclavicular; CC, coracoclavicular.

## Discussion

In this long-term follow-up of an unselected urban AC joint injury cohort, most respondents reported favourable shoulder function at a mean of 7.6 years after injury. Still, younger adults had significantly lower OSS scores than the normal population. We found no statistically significant differences in OSS between Rockwood types, or between grouped types I–II, and types III and V. However, the small numbers of Rockwood type V injuries resulted in imprecise estimates and limit conclusions regarding differences according to injury severity. Accordingly, these findings should not be interpreted as evidence that outcomes are equivalent across Rockwood categories. The CMS was relatively high and showed no significant differences between Rockwood type I and types II, III and V. There were 18 outliers in our study regarding OSS, and they experienced significantly more pain than the others. Degenerative findings, such as AC joint osteophytes or osteolysis, were common on follow-up radiographs but showed no association with OSS in multivariable regression analysis. This supports the view that radiographic pathology did not necessarily translate into functional impairment.

Our findings are consistent with previous long-term studies reporting generally favourable outcomes after non-operative management of Rockwood type I–III injuries.^47 48^ The present study adds data from a prospectively registered, unselected 1-year urban cohort rather than a cohort selected by injury grade or treatment. This design provides information on the relative distribution and long-term natural history of AC joint injuries encountered in routine emergency care. However, the predominance of low-grade injuries means that the principal contribution relates to the natural history of these injuries rather than comparative effectiveness of treatment for high-grade dislocations.

Several recent randomised controlled trials and systematic reviews have demonstrated that operative options, such as hook plate fixation or ligament reconstruction, do not yield better long-term outcomes than non-operative treatment.^5 6 15^ For example, Boström Windhamre *et al* reported no difference in clinical outcome at 2 year follow-up for Rockwood type III and V injuries treated with either hook plate fixation or physiotherapy.^15^ A meta-analysis by Chang *et al* and a multicentre trial by the Canadian Orthopaedic Trauma Society also concluded that operative treatment of acute high-grade AC dislocations offers no advantage over non-operative treatment in terms of pain, strength or patient satisfaction.^5 6^ Similarly, Haugaard *et al* reported that 91% of patients with Rockwood type III and V dislocations recovered well without surgery, with no significant difference in function between groups.^17^ Our findings are broadly consistent with these studies, although the small number of Rockwood type V injuries limits direct comparison. The absence of significant differences between injury types and the high mean OSS across the cohort suggest that long-term function remains largely intact, even in individuals with high-grade injuries managed non-operatively. These findings further support current recommendations that prioritise non-surgical treatment for Rockwood types I–III and selected type V cases.^1 3 4 11^ The absence of statistically significant differences should not be interpreted as evidence of equivalence between injury grades or treatment strategies. Decisions regarding surgery should continue to be based on the overall clinical presentation, persistent functional impairment, instability and current evidence.

While our study design did not allow for direct comparison with operative AC outcomes, the low rate of follow-up surgeries (3%) and favourable PROM scores indicate that most patients treated non-operatively do not need surgical intervention later. When we compare our cohort’s OSS scores to a normal population (Clement *et al*), we observe that younger adults, particularly males aged 20–40 and females aged 20–30, score significantly lower.^49^ This difference may reflect higher physical demands or greater functional expectations in this group, highlighting a potential subgroup where residual symptoms may have a greater impact.

### Clinical implications

In our study, 18 individuals were identified as outliers with an OSS below 26. They did not differ significantly from others in age or sex, but reported significantly more pain over the last 2 weeks before follow-up. A total of 12 patients underwent surgery in the shoulder region during this period, with only half undergoing an AC procedure. Given this and the fact that around half of shoulder injuries are soft-tissue injuries, especially in younger ages, this could be the problem for those with persistent pain, even though clinical and radiographic signs initially match an AC injury.^18^

The findings may reassure patients with AC joint injuries, as most respondents reported favourable long-term function. They also illustrate that radiographic abnormalities are not necessarily accompanied by poor patient-reported function. However, the study does not establish the comparative effectiveness of operative and non-operative treatment. Patients with persistent symptoms should undergo a comprehensive clinical assessment, including consideration of associated soft-tissue pathology, rather than having symptoms attributed solely to the radiographic appearance of the AC joint.^50^

### Limitations

This study has several limitations. First, it is a long-term follow-up study based on prospectively registered data, which limits our ability to establish causality. Second, the Rockwood classification used in this study depended on consensus interpretation, which, despite efforts to ensure reliability, could introduce classification bias. Furthermore, some patients might have sustained an extra soft tissue injury, which could affect the outcome. Third, although follow-up rates were high (84%), loss to follow-up and non-response may have resulted in selection bias. Recorded baseline characteristics and Rockwood distribution were generally similar between responders and non-responders, but the groups may have differed in unmeasured characteristics or long-term outcomes. Fourth, this study does not allow us to draw conclusions about the outcomes of type IV and VI injuries, as they were absent in the cohort. Fifth, the absence of a matched asymptomatic control group is a limitation. Although comparisons were made with normative population data, direct matching by age, sex and physical activity would have improved the interpretation of outcome scores. Another limitation is that the cohort comprised only ten respondents who had type V injuries. Consequently, comparisons involving Rockwood type V injuries had limited statistical power and wide uncertainty, and the absence of statistically significant differences should not be interpreted as equivalence. Finally, the effectiveness of commonly used measurements (OSS, QuickDASH, CMS) may be limited due to their lack of validation as tools for AC joint injuries.

Future research should include longitudinal studies of treated and untreated AC joint injuries using standardised imaging and functional assessments conducted at multiple time points to track recovery trajectories. Randomised controlled trials with long-term outcomes may identify, if any, subgroups that could benefit from surgical intervention. It is also essential to investigate predictors of poor outcomes in patients with persistent pain or dysfunction to guide individualised management.

## Conclusion

In this unselected urban cohort, most respondents reported favourable shoulder function at a mean follow-up of 7.6 years after initial non-operative management. We found no statistically significant differences in OSS across Rockwood types, although the small numbers of Rockwood type V injuries limit the precision and generalisability of these comparisons. Younger adults had slightly lower OSS scores compared with the uninjured normative population, but overall recovery was favourable. Radiographic degenerative changes were common but were not associated with patient-reported function. Persistent symptoms warrant individual clinical assessment, including consideration of associated shoulder pathology. The study does not establish comparative treatment effectiveness.

## Supplementary material

10.1136/bmjsem-2025-003183 online supplemental figure 1

10.1136/bmjsem-2025-003183 online supplemental figure 2

10.1136/bmjsem-2025-003183 online supplemental table 1

10.1136/bmjsem-2025-003183 online supplemental table 2

10.1136/bmjsem-2025-003183 online supplemental table 3

10.1136/bmjsem-2025-003183 online supplemental appendix 1

## Data Availability

Data are available upon reasonable request.
