# Supplementary material for: Long-term outcome of acromioclavicular joint injuries in an urban population
Source: BMJ Open Sport Exerc Med. 2026 Nov 10;12(4):e003183. doi: 10.1136/bmjsem-2025-003183 (PMC13756458; doi:10.1136/bmjsem-2025-003183)
Supplement: Supplementary file 1 [file bmjsem-12-4-s003.pdf]

Supplemental Table 1 Baseline characteristics of responders versus non-responders with acromioclavicular joint injury, number (%) unless otherwise stated.

	Total	Responders	Non-responders	p
N	287	240	47	
Age, years, median[a]	32.2 (24.5-44.4)	31.9 (24.4-44.2)	33.1 (25.6-47.0)	0.47
Male sex	234 (82)	198 (83)	36 (77)	0.34
NRS at injury[b] mean ± SD	5.7 ± 1.9	5.7 ± 1.9	5.9 ± 1.9	0.48
Sport injury	153 (53)	134 (56)	19 (40)	0.05
Right-sided AC joint injury	162 (56)	130 (54)	32 (68)	0.08

[a] Data presented as median (25th-75th percentile)
[b]Numeric Rating Scale (0-10) for pain, where 0 is no pain. At injury, as an indication of pain intensity.
