# Supplementary material for: Long-term outcome of acromioclavicular joint injuries in an urban population
Source: BMJ Open Sport Exerc Med. 2026 Nov 10;12(4):e003183. doi: 10.1136/bmjsem-2025-003183 (PMC13756458; doi:10.1136/bmjsem-2025-003183)
Supplement: Supplementary file 2 [file bmjsem-12-4-s004.pdf]

Supplemental Table 2 Numeric Rating Scale (NRS)[a,b] for pain in the injured acromioclavicular (AC) joint over the last 2 weeks prior to follow-up. Number (%).

	Rockwood[c] classification of AC joint injury				
	Type I	Type II	Type III	Type V	Total
NRS					
0	79 (53)	12 (52)	31 (55)	4 (40)	126 (54)
1	27 (18)	4 (17)	11 (20)	3 (30)	45 (19)
2	15 (10)	4 (17)	4 (7)	3 (30)	26 (11)
3	8 (5)	2 (9)	6 (11)	0 (0)	16 (7)
4	5 (3)	0 (0)	0 (0)	0 (0)	5 (2)
5	5 (3)	1 (4)	2 (4)	0 (0)	8 (3)
6	1 (1)	0 (0)	1 (2)	0 (0)	2 (1)
7	2 (1)	0 (0)	0 (0)	0 (0)	2 (1)
8	3 (2)	0 (0)	1 (2)	0 (0)	4 (2)
9	1 (1)	0 (0)	0 (0)	0 (0)	1 (0)
10	0 (0)	0 (0)	0 (0)	0 (0)	0 (0)
Total	146 (100)	23 (100)	56 (100)	10 (100)	235 (100)

[a] Fraenkel, L. Falzer, P. Fried, T. Kohler, M. Peters, E. Kerns, R. et.al. Measuring pain impact versus pain severity using a numeric rating scale. J Gen Intern Med. 2012 May;27(5):555-60. DOI: 10.1007/s11606-011-1926-z
[b] Numeric Rating Scale (0-10) for pain, where 0 is no pain.
[c] Rockwood classification from Type I-VI. No Type IV or VI were found in our material.
