# Supplementary material for: Long-term outcome of acromioclavicular joint injuries in an urban population
Source: BMJ Open Sport Exerc Med. 2026 Nov 10;12(4):e003183. doi: 10.1136/bmjsem-2025-003183 (PMC13756458; doi:10.1136/bmjsem-2025-003183)
Supplement: Supplementary file 3 [file bmjsem-12-4-s005.pdf]

Supplemental Table 3 Determinants of Oxford Shoulder Score, univariate and multivariate linear regression analysis (n=240).

		Univariable			Multivariable		
	N	coef.	95% CI	p	coef.	95% CI	p
Age at injury	240	-0.03	(-0.12 to 0.05)	0.414	-0.03	(-0.12 to 0.05)	0.430
Male sex	198	0.70	(-2.18 to 3.58)	0.631	0.51	(-2.54 to 3.57)	0.741
Left shoulder	110	1.06	(-1.13 to 3.25)	0.342	0.71	(-1.57 to 3.00)	0.539
Rockwood							
Type I	146	ref.			ref.		
Type II	23	3.27	(-0.55 to 7.09)	0.094	3.19	(-0.66 to 7.04)	0.104
Type III	56	0.84	(-1.84 to 3.52)	0.536	0.66	(-2.08 to 3.40)	0.637
Type V	10	0.19	(-5.38 to 5.76)	0.947	0.67	(-5.22 to 6.56)	0.824
Days to contact A&E[a]	240	-0.08	(-0.24 to 0.09)	0.349	-0.07	(-0.25 to 0.11)	0.462

[a]Days from injury to contact Accident & Emergency Department (A&E)
