# Supplementary material for: Long-term outcome of acromioclavicular joint injuries in an urban population
Source: BMJ Open Sport Exerc Med. 2026 Nov 10;12(4):e003183. doi: 10.1136/bmjsem-2025-003183 (PMC13756458; doi:10.1136/bmjsem-2025-003183)
Supplement: Supplementary file 4 [file bmjsem-12-4-s006.pdf]

Appendix A. Anamnestic questionnaires related to AC joint injury

Question	Response options / coding
Confirm shoulder with AC joint injury in 2013/2014	0 = Right 1 = Left
How satisfied are you with your shoulder today?	Continuous / ordinal scale 0 (worst)-10(best)
Do you have any other shoulder injury/pain other than the AC joint injury in the same shoulder?	1 = Yes 0 = No
Did this occur before or after the AC joint injury?	1 = Before 0 = After
Have you had surgery on the affected shoulder?	1 = Yes 0 = No
Have you had surgery for the AC joint injury?	1 = Yes 0 = No
When were you operated on for the AC joint injury?	Date (dd.mm.yyyy)
Have you sought other healthcare for the injury?	1 = Yes 0 = No
How many times have you sought other healthcare for the AC joint injury?	Numeric (count)
Have you smoked since the AC joint injury?	1 = Yes 0 = No
Do you currently smoke?	1 = Yes 0 = No
Do you use pain medication?	1 = Yes 0 = No
Do you use pain medication for the AC joint injury?	1 = Yes 0 = No
How often do you use pain medication?	1 = Daily 2 = More than 3 times per week<br>3 = 1–3 times per week 4 = Less often
